# Inflammation in Elite Athletes: A Review of Novel Factors, the Role of Microbiome, and Treatments for Performance Longevity

**DOI:** 10.7759/cureus.72720

**Published:** 2024-10-30

**Authors:** Elliot Dinetz, Nataliya Bocharova

**Affiliations:** 1 Integrative Medicine/Functional Medicine, University of Miami Miller School of Medicine, Miami, USA; 2 Internal Medicine, Pikeville Medical Center, Kentucky, USA

**Keywords:** athlete longevity, athletic performance, chronic inflammation, chronic injury, holistic approaches, injury prevention, microbiome and sport, sports injury prevention

## Abstract

Inflammation is a prevalent issue among athletes, especially those engaged in high-intensity sports, and can reduce the athlete's long-term potential. Over time, chronic inflammation impedes recovery, performance, and ultimately competitiveness, which is evident among some athletes over others. This article explores more novel concepts contributing to inflammation such as the microbiome and genetic predispositions, which are often overlooked in mainstream management, thereby allowing for more personalized therapies for athletes. Additionally, it introduces ways to address inflammation early to help prolong performance longevity and reduce the risk of serious injury holistically for the overall well-being of professional athletes.

## Introduction and background

Inflammation is the body's protective mechanism against pathogens, trauma, toxins, and infections [1]. However, chronic inflammation can significantly impact health and wellness. Currently, 125 million Americans suffer from chronic inflammation, which can be attributable to various causes, including unresolved infections, genetic predisposition, and immune dysregulation [2-4]. For athletes, chronic inflammation can drastically impact performance and longevity, explaining why some retire early while others continue playing into their 40s.Beyond pain and musculoskeletal injuries, different signs of chronic inflammation can help in earlier detection, which may help to save careers. Dermatological conditions, for example, are often a reflection of such inflammation from within [5,6]. Several studies have investigated the effects of inflammation in athletes, highlighting a plethora of risks and adaptive responses related to high-intensity sports in particular [7,8]. Endurance athletes, including cross-country skiers and marathon runners, have been found to have higher levels of inflammatory markers, with a nearly 60-fold increase compared to non-athletes [7,8].

Elevated levels of cytokines like interleukin-6 (IL-6) and tumor necrosis factor-alpha (TNF-alpha) have also been linked to psychological conditions, including mood disorders such as depression and anxiety. This connection highlights a bidirectional relationship between inflammation and mental health [9,10]. However, it is important to note that this topic falls outside the scope of this article. This article examines the multifactorial nature of chronic inflammation, focusing on overlooked, more novel factors such as the role of the gut microbiome, intestinal permeability, and genetic factors in inflammation and athletic performance. Additionally, it explores preventive and management strategies to enhance athlete health in a personalized, precise manner for career longevity.

## Review

Methods

This review was conducted through a comprehensive literature search and analysis of peer-reviewed articles and clinical studies. The primary focus of this article is to support the fact that microbiome is an overlooked factor in chronic inflammation in athletes. Furthermore, this critical juncture provides further opportunities for novel treatments. This review was conducted using electronic databases, such as PubMed and Google Scholar, covering the period from 1992 to 2024. The search was specifically focused on targeting the latest evidence on the role of microbiome in chronic inflammation in athletes and specific genetic markers of inflammation. Keywords such as "inflammation," "athletes," "microbiome," "markers of inflammation," "athlete longevity," “genetic markers of inflammation,” and "injury prevention" were used to ensure a thorough search of literature. Inclusion criteria involved systematic reviews, original research studies, book publications, data from the National Institutes of Health (NIH), and meta-analyses that focused on the relationship between inflammation in athletes, the role of the microbiome, specific genetic markers involved in inflammation, and novel therapeutic strategies in targeting inflammation. Both males and females were considered. Exclusion criteria included studies lacking full-text availability and publications in a language other than English. These findings were summarized to exhibit key mechanisms linking chronic inflammation to athletic performance and to highlight interventions aiding in enhancing athletes’ sports longevity. This is a narrative review, and the potential limitations may include selection bias. Furthermore, some newer studies in the field might have been missed due to the evolving nature of the subject.

Review

The gut microbiome, consisting of trillions of microorganisms, plays a crucial role in regulating inflammation and immune responses, impacting overall health and longevity [11,12]. It influences energy extraction, immune regulation, mucosal health, and brain function, all of which can affect an athlete’s success [13,14]. Exercise particularly influences the microbiome. While increasing exercise is healthy for this ecosystem, excessive or prolonged bouts may disrupt the balance between nutrients passing through the gut and potentially harmful substances, such as antigens, crossing the gut-blood barrier [15]. This supports why some high-endurance athletes end up with various complications ranging from autoimmune conditions to early heart disease [16,17]. Maintaining a balanced and diverse microbiome is crucial for preventing inflammation and supporting overall health [18,19].

Certain bacterial strains like *Lactobacillus *and *Bifidobacterium *may have anti-inflammatory effects, improving gut barrier integrity while also inhibiting harmful bacterial growth [20]. Athletes taking such probiotics have shown improved performance with reduced time to fatigue and decreased creatine kinase levels (CK) [21]. These particular strains of bacteria are typically seen in higher abundance naturally in youth and decrease with age, as does microbial diversity. Prebiotics, such as lactulose and inulin, among other fermented vegetables enhance beneficial gut microflora, fostering a diverse and abundant microbiome ecosystem. This has been shown to have an anti-inflammatory impact by decreasing TNF-alpha levels and increasing interleukin-10 (IL-10) [22]. Athletes may therefore support gut health by routinely consuming a fiber-rich diet consisting of fruits and vegetables, as well as probiotic-rich foods like yogurt and kefir, rather than solely relying on probiotics, which have limited total diversity with unknown long-term effects [15,23].

Dietary factors such as pro-inflammatory foods and seed oils damage the balance of the microbiome on the other hand. Pro-inflammatory foods include added refined sugars, saturated and trans fats, and processed seed oils high in omega-6 fatty acids. Unfortunately, these components are prevalent in numerous food items, including those marketed as health food bars. This makes it difficult for athletes and health-conscious individuals to distinguish healthy options in a saturated market. Seed oils, such as soybean, corn, canola, and sunflower oil, can disrupt the balance between omega-3 and omega-6 fatty acids, leading to increased inflammation and permeability in the gastrointestinal tract [24]. These are often extracted from genetically modified organisms (GMOs) and crops loaded with pesticides known to damage the gut microbiome as well. Consumption of seed oils has been linked to higher risks of numerous chronic diseases [25,26]. In the long term, this likely affects athletic performance on many levels. Athletes, alongside their teams, may benefit from diligently avoiding these ingredients and sticking to whole food products. Clean substitutes made with olive oil or avocado oil, if any added oils were to be used, provide less inflammatory substitutes.

The skin acts like a reflection of gut health as we see conditions like atopic dermatitis and eczema flare from permeability and dysbiosis [6,27,28]. Further, vitiligo and cystic acne are not uncommon among some top athletes, ranging from recent Olympians to professional tennis and soccer players, suggesting that such disturbances in the gut microbiome and permeability are not being properly addressed. Both skin cells and gut cells originate from neural crest-derived precursors embryonically. Although they have different functions, both are involved in barrier protective functions with distinct microbiomes. Research has shown disparities in gut microbial diversity between individuals with acne and those without [28,29]. In elite athletes, strenuous activities further increase gut permeability via prolonged stress in the fight-or-flight state, fueling the flames of such systemic conditions [30]. Dermatological issues like cystic acne can be early warning signs of underlying health issues, even in athletes who are considered to be in great physical shape and are assumed to be healthy. This should be a foreshadowing to trainers and athletes alike to know that they may have unmitigated inflammation to further explore and address.

Intense physical activity, which many high-performance athletes are subject to, especially when prolonged, can lead to increased intestinal permeability, which is commonly referred to as “leaky gut.” This leads to systemic inflammation and hypoxia due to reduced splanchnic blood flow [31,32]. A leaky gut affects the microbiome by influencing its composition, function, and overall balance. When the gut barrier is compromised, it can alter the local environment in the intestines, leading to dysbiosis. Additionally, when lipopolysaccharides (LPS), toxins, and undigested food particles enter the bloodstream, the immune system becomes activated. This can lead to an inflammatory response affecting the gut microbiome. Excessive exercise not only changes the microbiome but also keeps the body in a prolonged sympathetic state in sports like cycling and marathon running. This stress can damage the cell lining of the gut, leading to long-term health issues and noticeable symptoms over time. While degeneration of the musculoskeletal system is the primary focus when it comes to injury, everything from mood disorders to dermatological inflammatory conditions may be a reflection of this gut-immune response. It is therefore likely that any processed foods, whether intentionally consumed or not, may further fuel chronic subclinical inflammation in metabolically healthy athletes [33,34].

Genome-wide association studies provide a significant amount of literature supporting genetic polymorphisms and their impact. While we are aware of these associations today, they may be also limited by several other factors including metabolomics, transcriptomic, and epigenetic effects on gene expression.By understanding their mechanisms of action, these pro-inflammatory gene markers provide valuable insights into potential risks and clues that individuals may be experiencing inflammatory issues. Gene polymorphisms are associated with increased inflammation propensity, subsequent systemic inflammation, and musculoskeletal damage.** **Single nucleotide polymorphisms such as IL-6, TNF-alpha, and nuclear factor-kappa B1 (NFKB1) genes are associated with greater muscle and tissue damage, requiring longer recovery from exercise [35-37]. Genetic profiling can help identify potential genetic issues and allow for personalized training and treatment strategies on a case-by-case basis. For example, those who are found to have such variants, regardless of exercise tolerance, should organize a tailored exercise approach around an anti-inflammatory lifestyle, which we will discuss further. Understanding how an individual is likely to respond to a specific type of exercise can enable coaches and practitioners to tailor exercise training for their athletes or patients. This personalized approach not only maximizes recovery and adaptation but also minimizes the risk of overtraining.

An effective approach to managing persistent inflammation in elite athletes should combine dietary strategies, targeted supplementation, and training based on individual needs to enhance performance and aid in recovery. Curcumin, found in turmeric, promotes beneficial bacterial growth [38], reduces inflammation in the gut [21], enhances gut bacterial function [39], and modulates gut microbial composition [38]. Curcumin has several mechanisms by which it mitigates inflammatory processes. It works by inhibiting NFKB, preventing it from entering the nucleus, and inducing inflammatory gene transcription [40]. Curcumin also reduces the production of pro-inflammatory cytokines such as IL-6, TNF-alpha, and C-reactive protein (CRP) 7. Therefore, athletes with single nucleotide polymorphisms in genes, such as IL-6, TNF-alpha, and NFKB, which predispose them to higher levels of inflammation could particularly benefit from curcumin’s anti-inflammatory properties. Moreover, for athletes with inflammation originating from gut microbiome-immune responses, curcumin may be very helpful in mitigating this cascade. Curcumin has demonstrated benefits for athletes by reducing exercise-induced damage at doses starting from 500 mg, with its safety well-established up to 12 grams, where only mild side effects have been reported [41,42]. With this understanding, athletes' regimens may be titrated based on a personalized approach to address inflammatory symptoms and track objective markers. This may reduce system-wide inflammation over time and maximize body systems performance, translating to longevity in the game.

Omega-3 fatty acids, such as eicosapentaenoic acid (EPA) and docosahexaenoic acid (DHA) also have anti-inflammatory effects, especially when maintaining ratios under 5:1, which can reduce the inflammatory cascade. In addition to curcumin, they may be used to target genetic predispositions to inflammation in high-endurance athletes who are exposed for longer periods to inflammation [43-45]. Glutamine supplementation strengthens the immune system while improving intestinal barrier function [46,47]. Moreover, supplementing with glutamine may be appropriate for all high-endurance athletes. Improvements in intestinal barrier function and mortality benefits in critically ill individuals have been documented at doses above 30 grams per day. This likely improves the microbiome-immune balance in prolonged sympathetic states. L-glutamine has been shown to help maintain gut integrity and prevent LPS-induced inflammation, making it essential for athletes undergoing significant intensity and prolonged training [48,49]. Antioxidant-rich foods and fermented dairy products are further shown to reduce inflammation and enhance performance among athletes [50-52]. Together, these ingredients may have additive benefits for future studies to examine another key to longevity, while each can lower insulin resistance.

A balanced microbiome-friendly diet is essential for athletes as the mainstay of lifestyle interventions [38]. Whole foods support a healthier microbiome while offering better nutrient bioavailability. Supplements can be used to address various situations, such as deficiencies and genetic variants, and further enhance performance and recovery by reducing inflammation [13,21]. Rather than prioritizing one approach over another, a combined method may yield the best results in maintaining long-term health and peak athletic performance.

Newer antioxidants for enhanced performance and recovery include hydrogen-rich water (HRW). This has been shown to reduce inflammation rapidly while promoting recovery by neutralizing free radicals [53]. Human trials have shown that this is effective in alleviating sports-related soft tissue injuries with 2 g/day and also helpful in dermatological conditions [53]. HRW may, therefore, be a mainstay for athletes with a predisposition to inflammation, prolonged sympathetic activation, high-endurance activities, and/or higher-impact sports or training, although more studies are required to evaluate its full impact.

Pre-workouts often contain beetroot juice with ingredients such as L-citrulline and L-arginine. These enhance nitric oxide production and subsequent blood flow, which are effective for cardiopulmonary performance at doses of 400-500 mg/day. This makes them a great option both before and potentially after long bouts of endurance exercise [54]. For athletes, including long-distance or endurance athletes, this could be key alongside branched-chain amino acids (BCAAs), which reduce muscle damage and promote protein synthesis, thereby aiding in recovery and performance [55,56].

Avoiding GMO gluten and incorporating vitamin E mixed tocopherol supplementation further support intestinal health and cell membrane integrity including the mitochondria [57-59]. Understanding these more novel integrative approaches along with personalized strategies can likely enhance overall health in an athlete. Utilizing genetic screening for a precision approach may help as a logical strategy that should be incorporated in all collegiate and professional athletes now, given that such testing is more readily available through functional and sports medicine physicians.

## Conclusions

Inflammation from sports and environmental exposures poses a significant challenge to athletes, affecting not only their performance longevity but also their overall health. Effective management of inflammation through diet, supplements, and personalized strategies is crucial for optimizing athletic performance and reducing the risk of injuries, considering the microbiome as an epicenter for much inflammation. Incorporating prebiotic and probiotic whole foods alongside pharmaceutical-grade supplements tailored to each athlete‘s unique genetic and clinical needs can enhance sports medicine and maximize athletic longevity. Athletes should collaborate with healthcare professionals and their teams to develop the most personalized interventions. This includes a thorough physical examination to identify all signs of chronic ongoing inflammation and awareness of intestinal permeability, which may be potentially at the root of it. By adopting a comprehensive strategy, athletes can not only unlock their full potential and ensure long-term success in their sports but also secure their future.
